# Adolescents at risk of self-harm in Ghana: a qualitative interview study exploring the views and experiences of key adult informants

**DOI:** 10.1186/s12888-020-02718-6

**Published:** 2020-06-16

**Authors:** Emmanuel N-B. Quarshie, Mitch G. Waterman, Allan O. House

**Affiliations:** 1 Department of Psychology, University of Ghana, P.O. Box LG 84, Legon, Accra, Ghana; 2 School of Psychology, University of Leeds, Leeds, UK; 3 Leeds Institute of Health Sciences, University of Leeds, Leeds, UK

**Keywords:** Adolescents, Ghana, Resilience, Rhetorical distancing, Self-harm, Street-connected adolescents, Sub-Saharan Africa, Suicide, Teachers, Social workers

## Abstract

**Background:**

In Ghana, rates of self-harm in young people are as high as they are in high income countries. Self-reported interpersonal, familial and societal stressors form the most important background, and self-harm is seen by young people as a way of responding to that stress. In the present study, we obtained the views of key adult informants about self-harm among adolescents in Ghana – what they thought as possible reasons for self-harm in young people and what actions might be needed at an individual or population level to respond to the problem.

**Methods:**

We interviewed face-to-face 11 adults, using a semi-structured interview guide. We used an experiential thematic analysis technique to analyse the transcribed interviews.

**Results:**

The analysis identified five themes: “underestimating the prevalence of self-harm in adolescents”, “life on the streets makes self-harm less likely”, “self-harm in adolescents is socially and psychologically understandable”, “ambivalence about responding to adolescent self-harm”, and “few immediate opportunities for self-harm prevention in Ghana”. Adolescent self-harm was acknowledged but its scale was underestimated. The participants offered explanations for adolescent self-harm in social and psychological terms that are recognisable from accounts in high income countries. Low rates among street-connected young people were explained by their overarching orientation for survival. Participants agreed that identification was important, but they expressed a sense of inadequacy in identifying and supporting adolescents at risk of self-harm. Again, the participants agreed that self-harm in adolescents should be prevented, but they recognised that relevant policies were not in place or if there were policies they were not implemented – mental health and self-harm were not high on public or political priorities.

**Conclusions:**

The adults we interviewed about young people who self-harm see themselves as having a role in identifying adolescents at risk of self-harm and see the organisations in which they work as having a role in responding to individual young people in need. These are encouraging findings that point to at least one strand of a policy in Ghana for addressing the problem of self-harm in young people.

## Background

There is little consensus in the literature about the definition of self-harm. This study follows the definition provided by the World Health Organisation (WHO): “an act with non-fatal outcome in which an individual deliberately initiates a non-habitual behaviour, that without intervention from others will cause self-harm, or deliberately ingests a substance in excess of the prescribed or generally recognised therapeutic dosage, and which is aimed at realising changes that the person desires via the actual or expected physical consequences” [1, 2]. Self-harm among young people is recognised as a public mental health problem associated with several negative outcomes including suicide [2, 3]. Much of our understanding is based on literature from countries in Europe (particularly the UK), North America, and Oceania; by comparison little is known about self-harm in young people in sub-Saharan Africa and other low- and middle-income contexts [4].

Our recent systematic review found a 12-month prevalence of 16.9% for self-reported self-harm among young people in sub-Saharan Africa [5], which is comparable to what is reported in high-income countries [6, 7]. In sub-Saharan Africa, factors identified as associated with self-harm include depression, hopelessness, psychiatric illness, conflict with parents, abuse and violence (sexual, physical and emotional), schoolwork problems, romantic relationship difficulties, and lack of social support [5].

In a cross-sectional questionnaire survey undertaken in the Greater Accra region of Ghana, we also identified levels of self-reported self-harm that are similar to those reported from high income countries (Quarshie ENB, Shuweihdi F, Waterman MG, House AO. Self-harm among in-school and street-connected adolescents in Ghana: a cross-sectional survey in the Greater Accra region, submitted). Rates of self-harm were substantially lower in street-connected adolescents^1^ than they were in those young people who were in school and living at home, despite the former group reporting more social and personal adversity. To explore these findings further we have conducted an interview study of adolescents in Accra: the participants reported interpersonal, familial and societal stressors as the most important background to their troubles, and described self-harm as a way of responding to that stress [8]. Street-connected adolescents were apparently more aware of the potential negative consequences of self-harm, such as incapacitation, injury or death.

To complement these studies, we wanted to obtain the views of adults who work with, live with or have a public responsibility for adolescents (with a self-harm history) in Ghana – what they thought of the social phenomenon, about possible reasons for self-harm in young people and about actions that might be needed at an individual or population level to respond to the problem as they saw it. Such research is needed in African countries to explore the personal thoughts, feelings, motivations of key informants and their socio-cultural context, as they describe and explain pathways to self-harm [9]. In Ghana, self-harm is highly stigmatised [10], as the behaviour is morally taboo [11, 12], religiously considered as a sin [13], and legally criminalised [14]: persons found guilty under the law have been given hefty fines or in some instances jailed [15].

## Aims and objectives

We sought to explore the perspectives of adults whose work is relevant to, or who live with, adolescents at risk of self-harm. Our research questions were: (i) How did these adults perceive the public health problem of self-harm in adolescents? (ii) How do they respond to individual episodes of self-harm in adolescents? (iii) What responses do participants offer to the question, “How can self-harm in adolescents be prevented in Ghana?”

## Methods

### Design and setting

We conducted a qualitative cross-sectional observational study involving one-to-one semi-structured interviews in the Greater Accra region of Ghana [16]. The data collection took place between August 2017 and April 2018.

### Sample

For the present study, we used purposive sampling to identify and recruit key adult informants [16]. At the time of administration of the questionnaire we approached face-to-face members of staff (heads of school, teachers, and school counsellors) in the schools where our previous questionnaire survey was conducted, and invited face-to-face members of staff (head of charity facility, and street social workers) at the charity facilities where the questionnaire survey was conducted with the street-connected adolescents. We invited by telephone five of the parents who provided consent for their underage wards to participate in our previous survey and qualitative interview studies with adolescents [8]. We approached by letters two government representatives with public roles in key departments that involve policy formulation and implementation regarding child and adolescent welfare and education, to discuss the purpose of the study and invited them to participate in the interview.

In all, we invited 26 potential participants, of whom 11 participated in this study: one head of a school, two teachers, a school counsellor, two parents, one head of a charity facility, two street social workers, and two government representatives (one each from the Ghana Education Service and the Department of Social Welfare).

The participants comprised five females and six males, aged between 30 and 55 years (mean = 45 years). All self-identified as Christian and employed. Each of them had a tertiary educational background, with number of years in current position at work ranging between one and 23 years (mean = 11 years).

### Interview

A semi-structured interview protocol was made up of three parts. In the first part each participant was asked in an open way to provide a description of their experience of self-harm and general view about self-harm in Ghana, including its prevalence. The second part of the interview involved questions probing relevant details that were not sufficiently addressed in the responses to the open questions. We asked about the respondents’ views on individual reasons for self-harm, based upon their own experience of encountering and responding to incidents. Finally, we asked for respondents’ views about what actions should be taken to respond to self-harm – either at an individual or population (public health) level.

All the interviews were audio recorded and transcribed by the interviewer (EQ) who also made field notes during and immediately after each interview to provide context for the transcripts. EQ is a Ghanaian and a trained Community Psychologist, with several years of experience in community research and mental health promotion in Ghana. EQ’s ‘insider’ status facilitated rapport in conducting the interviews [17]. Eight participants opted for the interview to be conducted in their office at their workplace, two participants (school staff) were interviewed in the empty library of their school, and one participant chose to be interviewed in the study of her home. On average each interview lasted 87 min (range 63–105 min); no follow-up or repeat interview was conducted.

### Analysis

The experiential thematic analysis technique was used to analyse the transcribed interviews. This technique is a variant of thematic analysis that focuses on the participants’ standpoint [18]. We followed a 6-step approach to analyse the transcribed interviews: familiarising ourselves with the data, generating initial codes, searching for themes, reviewing themes, defining and naming themes, and producing the report [18–20]. We discussed the emergent themes until consensus was reached. Critical comments from the co-authors (who are international researchers but ‘outsiders’ to the Ghanaian culture) ensured that the first author’s personal biases and assumptions, as an ‘insider’ of the socio-cultural context of Ghana, did not blur the interpretation of the data and equally important context relevant factors were not overlooked in the analysis and interpretation of the data. This helped to preserve the reliability and validity of our findings. Due to the busy nature of the participants’ work and the sensitivity of self-harm in the Ghanaian society, it was not possible to return to the participants to validate our findings.

## Results

The participants’ views and accounts were based on their experiences of previous encounters with adolescents who had self-harmed or were at risk of self-harm; one participant (F4) made additional inferences from her personal adolescent self-harm experience. Our main findings were organised around five themes: “underestimating the prevalence of self-harm in adolescents”, “life on the streets makes self-harm less likely”, “self-harm in adolescents is socially and psychologically understandable”, “ambivalence about responding to adolescent self-harm”, and “few immediate opportunities for self-harm prevention in Ghana”.

### Underestimating the prevalence of self-harm in adolescents

At the introductory phase of each interview, we asked about the participant’s awareness of the extent of the phenomenon of adolescent self-harm in Ghana generally, and more specifically within the participant’s institution (among students in second cycle schools or street-connected young people attending charity facility) or family (adolescent children, in the case of parents). There was a general agreement from our participants that self-harm among adolescents is a recent phenomenon in Ghana, and often the behaviour is hidden. However, the participants had differing views as to the exact extent of the phenomenon and the potential factors that could account for the differences in rates between in-school and street-connected adolescents. Four subthemes emerged: “adolescent self-harm as a hidden behaviour”, “rhetorical distancing”, “failure to recognise the extent of adolescent self-harm” and “self-harm rates are lower among street-connected adolescents”.

- “If the adolescent dies, then we hear of suicide”: Adolescent self-harm as a hidden behaviour

All the adults agreed that the true picture is difficult to gauge because adolescents who self-harm often hide the behaviour:

*[…] the matter of self-harm, to me, the adolescent does it in secret. If the adolescent dies, then we hear of suicide. But the real self-harm behaviour is a secret thing that you don’t really get to know about, unless you make a very close observation of the adolescent. Some cut themselves on the laps or belly, and you can’t see [it] because the school uniform covers the cuts or burnt or whatever. So, unless they tell you or you see that their behaviour has changed […] (M2, male, Ghana Education Service representative).*

b. “No child of mine has ever or will ever self-harm…”: Rhetorical distancing

The respondents, particularly the parents and school staff, provided descriptions that sought to idealise and project their school or home as being “free from” adolescent self-harm.

*No child of mine has ever or will ever self-harm or even think of suicide. I say this with confidence because you see, as parents, we have to raise our children well…. When they [my children] were much younger, if there was anything they needed to know, I told them. … […] I’m closer to my children […] Their father also does the same thing. (F5, female, parent).*

Coincidentally and unknown to F5, her daughter had also participated in our research, sharing her personal experience of self-harm [8]. Her narrative sharply contrasted her mother’s view that “no child of mine has ever or will ever self-harm”. Also, the daughter implied in her narrative that her mother controlled their social relationships in the neighbourhood, a view which contradicted the seeming non-authoritarian parenting style offered by her mother [8].

c. “In this school, self-harm is rare”: Failure to recognise the extent of adolescent self-harm

While not exhibiting the downplaying seen in rhetorical distancing, some school staff failed to recognise the extent of self-harm among the adolescents in their schools.

*In this school, self-harm is rare. [Um], for example, if school is in full session, we have about 3000 students in total. Now, within the whole academic year, you can have just one case of self-harm. So, it is not something that is frequent in this school; hardly will you hear about such things here, it’s not common (F3, female, school counsellor).*

Notably, a total of 121 randomly selected students in her (F3) school participated in the self-report survey. Of this sample, 21 (17%) reported that they had self-harmed during the previous 12 months. Interestingly, later in the interview with this participant (F3), we asked about the challenges she faces in providing support to students who self-harm in her school. Her response told a different story:

*[…] Luckily, at our [counselling] unit, we are attached to the psychiatric hospital, so we are able to refer more severe cases to them [psychiatric hospital] […] If it’s not for that arrangement, um, we would be overwhelmed, because every term, we get cases of students who self-harm in one way or another and for various reasons.*

The charity facility staff also acknowledged the topical nature of self-harm among adolescents in Ghana, but suggested that, compared to in-school adolescents, self-harm might be less frequent among street-connected adolescents, at the same time acknowledging that it might be difficult to verify.

*[…] self-harm is not very frequent and not very clear or visible in the streets. You can see a boy or a girl who has unusual bruises or cuts on his/her hand, but he can tell you that he was attacked or beaten by someone, and because these young people are often exposed to these kinds of physical abuses in the streets, you won’t doubt his story. […] Although I have heard that some young people in the street intentionally harm themselves …, I have not directly encountered one myself in the streets. But in the drop-in centre here, I have seen just a few (M1, male, street social worker).*

**“What they think about is survival”: life on the streets makes self-harm less likely.**

We explored the finding of the questionnaire survey that, compared to in-school adolescents, the prevalence estimate of self-harm was low among the street-connected adolescents. Interestingly, even though some adults responded by attempting to compare and contrast the life circumstances of the two adolescent groups, virtually all tended towards emphasising factors that could account for the low estimates among the street-connected adolescents, rather than explaining the high estimates among in-school adolescents.

The responses suggested that street-connected young people’s overarching orientation for survival on the streets makes them less likely to choose self-harm. Street-connected young people strive on a daily basis to meet their basic needs of food, clothing, and sleeping place; their primary drive is to find self-support strategies and engage in activities to help them cope with their daily needs and adversities in order to survive.

*On the streets, um, there is nothing like rights, unlike the home where a child learns at school that they have the right to food, shelter, education and all that from their parents. It doesn’t happen on the streets. You have to struggle every day to survive, um, so where is the chance to even think of harming yourself? Of course, as humans, a street child may have their low moments when they are not happy, but I think street children are stronger (F4, female, teacher).*

Additionally, three sub-themes helped to explain the overarching theme “life on the streets makes self-harm less likely”: a) street life generates resilience, b) street-connected young people are free spirited, and c) street-connected young people have access to drugs.

#### a. Street life generates resilience

These views seem to suggest that daily struggles for survival make street-connected young people more resilient and less likely to choose self-harm than young people who live in intact families.

In the street context, there are no parents or adult figures to provide guidance and the material needs of young people; street-based families are poor and often unavailable to provide meaningful parental supervision and emotional support to young people. The implication is that street-connected young people must be self-reliant to make decisions and provide for themselves support that ordinarily a parent figure must make or provide. According to the participants, street-connected young people take on these adult roles at a very young age, making them self-reliant and 'mature' early, and this possibly also partly account for the low rates of self-harm among this group of young people.

*You see, the street life makes them mature early. I’m saying this because what a 13-year old boy in the street can say and do, a 13-year old boy in school and at home cannot say or do. In the street, you need to be strong; you have to work, you have to manage your money, and you have to think about what you will eat tomorrow. So, these things make them develop a strong sense of being responsible for yourself every day. But generally, those at home [in-school adolescents] have parents or other relatives who provide for them, so at the least thing they may think [about] or want to self-harm (M1, male, street social worker).*

#### b. Street-connected young people are free spirited

Here also, the participants argued that street-connected adolescents have low rates of self-harm because their motivation to survive on the streets makes them free-spirited: they are independent and have unrestricted freedom and total control over their resources and social relationships.

*On the street, they [young people] have more time to do everything they want, they have friends who are like them and who understand them. They have freedom. They work to make money; they have the money. [um] So they’re able to buy whatever they want, and they spend their time anyhow they want (M6, male, representative, Department of Social Welfare).*

#### c. Street-connected young people have access to drugs

To survive the various forms of distress on the streets, many street-connected young people use drugs, access to which is unfettered in the street context. The participants posited that whereas many in-school adolescents choose self-harm to cope with their distress, many street-connected adolescents resort to other equally unhealthy behavioural options, mainly alcohol and substance misuse.

*[…] children who are in school may choose self-harm when they are not happy, but on the streets, the street children choose drugs and other substances. Many of the children who come here [charity facility] have this problem. Um, for instance, now they buy tramadol and add it to energy drinks in high quantities and drink, others also smoke marijuana. They say that they do it because it makes them happy, or so that they can get more energy to work [in order] to make more money, and they’re also doing it in order that they can sleep well. […] But the thing is that abusing these drugs over time can be harmful and even kill them (F1, female, head of charity facility).*

### Self-harm is socially and psychologically understandable

This major theme describes the adults’ views about the key circumstances that lead up to self-harm in adolescents in Ghana – seen as mainly due to acute negative emotions resulting from multiple negative events and difficulties: conflict with parents, child powerlessness in the family, harsh parental control over adolescent relationships, family poverty, intra-familial sexual abuse, harsh punishment, poverty, death of significant other adult, lack of social support, diabolical manipulations,^2^ bullying victimisation, accusations and social taunting, failure to meet pressure to perform academically, poor academic performance, and untreated mental health problems.

*[…] some of these students are struggling emotionally to fit in and be accepted among their peers [...] Many adolescents are on social media and they get trolled and bullied. We see instances where students upload the naked pictures or videos of others […] Sometimes too, some girls are called ‘ashawo’*^3^*or witches, not only by their peers, but by their family members. When I was growing up as a teenager, I was the only slim one in my family. I remember sometimes they would make fun of me – my siblings and even my parents, at one point or the other. It got to a time I was struggling to accept myself; yeah, I remember very well that my mom used to call me ‘ghost’. And to be honest, there were few times I thought of committing suicide, yeah, it was a struggle (F4, female, teacher).*

*Two years ago, we had a girl [in this school] who took overdose of Ibuprofen because her failure results were pasted [on the school’s noticeboard] and her classmates and even her juniors got to know about it [...] So, I advised the headmistress against pasting the results of students on the noticeboard. If you put the results out there, mentally, you put needless pressure on the students, and it can be shameful and embarrassing for the less performing students. Three years ago, a student at the [mentioned name of*^4^*] university committed suicide because his failure results were pasted on the noticeboard of the university. But I don’t think the problem was the failure* per se*, rather I think the pressure and the shame and the embarrassment that came with the failure were what triggered the suicide (F3, female, school counsellor).*

*[…] some of the students work. After closing from school, they go to sell ‘pure water’,* 5*food and drinks, and others are shopkeepers at home. These students work every day to generate income to support their families, yet some of them come to school with little or no money at all. As a teacher, you can see the child is hungry and so you’ll give them money to buy food […] Most of them come from poor families with six or more children and a single parent, or even if both parents, they are not gainfully employed […] How can they provide the needs of the child? How can the child be happy? Many of these students are not enjoying their childhood, they are hopeless. So why won’t they engage in self-harm or even commit suicide to end it all? (M4, male, teacher).*

*[…] The girl [my niece] combined different kinds of medicines [tablets] and drank. She wanted to commit suicide because her stepfather was sexually molesting her […] But whenever it happened, and she told her mother about it, she [her mother] didn’t believe her […] At the hospital, she [my niece] told me everything, so I called the police and the man [stepfather] was arrested […] He is still in jail (M3, male, parent).*

We were struck that these accounts often failed to explain the adolescents’ rationale for choosing self-harm out of other possible behavioural options (e.g., running away from home, reporting the abusive significant other adult to the police, seeking help, or using other negative coping strategies). When explanations were offered, they were usually in stereotypical terms: a) “self-harm as a girl thing”, b) “self-harm as a coping mechanism”, c) “self-harm to have a voice”, and d) “self-harm as a sensation-seeking behaviour”.

- “…girls brood and blame themselves…, boys fight back”: Self-harm as a girl thing

In characterising self-harm in adolescents, many of the adult stakeholders reported that self-harm is gender patterned, often with more females, than males, engaging in the behaviour. They reflected that compared to adolescent boys, adolescent girls tend to have internalising tendencies, face multiple unmet needs, and experience various emotional challenges, which lead to increased likelihood of self-harm.

*[…] most of the time, it is more girls than boys who do it [self-harm], but when it comes to harming another person then it is the boys who do that more. I don’t mean to discount the fact that boys also engage in self-harm, they also do […] The thing is that, usually, most girls brood and blame themselves when bad stuff happen to them, they don’t fight back, but boys fight back (F4, female, teacher).*

b. “To…forget about the pain in her heart for a while”: Self-harm as a coping mechanism

Some participants suggested that some adolescents self-harm in order to reduce the discomfort associated with emotional distress; their skills to solve problems rationally are not fully formed or may be absent.

*When school resumed last term, a girl came to me with an issue […] Her boyfriend dumped her and she had a broken-heart […] She said that she sometimes felt like dead, and anytime she felt that way, she would use a divider to prick her thigh to keep her alert or to make her forget about the pain in her heart for a while […] They [adolescents] engage in self-harm because of immaturity, and they’ve not yet developed proper problem-solving skills, you see? (M4, male, teacher).*

c. *“He didn’t like any of those impositions”: Self-harm to have a voice*

The school staff reflected that some adolescents use self-harm as means of having their voice heard and asserting their autonomy in their families. They argued that for adolescents who come from families where they are not allowed to participate in making decisions that affect them, self-harm or the threat of self-harm forces such families to make room to accommodate the views of the adolescents. For example,

*Two years ago, I had this student who attempted to stab himself at home. He said he was ‘bundled’ and brought to the boarding house. Also, his parents said he had to read medicine at the university to become a doctor in future, so he must do science here [in the senior high school]. He didn’t like any of those impositions. So, you see, he attempted to stab himself in order to be heard by his parents (F3, female, school counsellor).*

d. “…just to see how it feels”: Self-harm as a sensation-seeking behaviour

The participants also characterised self-harm in adolescents as sensation-seeking. They argued that some adolescents learn to self-harm through watching self-harm scenes on television and seek ways to experiment it.

*[…] we were discussing something in class, and she [a student] said that there is something that fascinates her. Whenever she’s watching TV, and someone gets upset and they take plates and angrily crash it to the floor or the person kicks or punches a wall; she also feels like doing the same. She wants to do same just to see how it feels. And these days self-harm and suicide are shown in films, so in the same way, some adolescents who see these films would also want to try it out, when they have emotional problems (F3, female, school counsellor).*

### Ambivalence about responding to adolescent self-harm

Three sub-themes emerged to elucidate the major theme of how the adult stakeholders respond to adolescent self-harm: a) “uncovering self-harm in adolescents”, b) “feeling inadequate to offer support”, and c) “redefining teachers’ roles”.

- *Uncovering self-harm in adolescents*

In both the schools and charity facilities, the participants indicated that most adolescents who self-harm do not normally voluntarily report or seek help from them (teachers, counsellors or social care professionals), and families rarely report cases of self-harm of their adolescent children to the school or charity facility. The school staff described three strategies they use concurrently or in isolation to identifying distress or behaviours that could potentially trigger self-harm or to identify adolescents who had self-harmed recently or were experiencing a self-harm crisis: observation for signs of distress, giving out emotive composition exercises and picking up elicited emotional responses, and class-based guidance talks followed by discussion.

At the charity facilities, the participants reported that while most street-connected adolescents share their problems and challenges with the members of staff, the adolescents who self-harm do not voluntarily seek help or where they do, they lie about the true cause of their injuries. Therefore, the charity facility staff tend to use unobtrusive monitoring as a strategy to help them identify self-harm in the adolescents.

b. *“You don’t even know of any first aid…”: Feeling inadequate to offer support*

All the adult participants reported that they did not have the right knowledge and lacked any professional training to enable them to provide meaningful support to adolescents who self-harm. Even so, only the head of charity facility and the school counsellor indicated that they made referrals to mental health professionals.

Teachers occupy a front-line position for both the identification of self-harm in adolescents and as the first point of contact for support by adolescents who self-harm, but the lack of an institutional protocol to guide the handling of adolescent self-harm seems to worsen individual teachers’ sense of incompetence and lack of confidence in offering support to adolescents who self-harm. For example,

*[…] There are no guidelines telling you what steps to follow. So, all you can do is to take the student to the headmaster or assistant headmistress, but what if these bosses are not available? The other thing is that, sometimes the issue worrying the student is so confidential and they trust you not to tell anybody, but here is the case you [as a teacher] also don’t know what to do; you don’t even know of any first aid to give the student (M4, male, teacher).*

This view was also shared by the head of charity facility,

*You’d usually read a general thing like, “ensure that the children are psychologically and mentally well”. I mean, this is very vague, because there is nothing about what specific actions to take to ensure that these young people are mentally and psychologically well […] I know schools also don’t have anything on student mental health. So, we need something more concrete from the Department of Social Welfare (F2, female, head of charity facility).*

c. *“We have to be both teachers and parents…”: Redefining teachers’ roles*

The school staff argued that, in order to make teachers’ contributions more meaningful in responding to adolescent self-harm, a shift in the traditional definition of teachers’ work might be needed. They suggested that the restriction of teachers’ role to academic progress and success of students might require a change to allow teachers to also provide social care and emotional support for the students they teach.

*[…] most teachers are parents too, but when we [teachers] come to school we tend to focus only on classroom progress and success in exams. I think this has to change; we have to combine both. We have to be both teachers and parents when we are in school, because these students need us to support them emotionally too […] They face too many challenges beyond the routine schoolwork of reading, writing and arithmetic […] (F4, female, teacher).*

### Few immediate opportunities for self-harm prevention in Ghana

All the participants agreed that self-harm in adolescents in Ghana could and should be prevented, as self-harm threatens the life and future of young people and affects their families and others around them. An obvious example was that adolescents experiencing emotional challenges (including self-harm crisis) should be able to seek help by talking to someone trustworthy. For instance,

*[…] Young people must talk to someone, when they [young people] are going through challenges like emotional problems or difficulties at home or when they have problems with their peers or school. They have to talk to a person they trust. If you cannot talk to your parent or an older sibling, try your teacher or your pastor or imam. If you have a problem and you don’t talk to someone about it, no one can help you (F1, female, social worker).*

A second suggestion was that the Ghana Education Service (GES) should consider including specific topics related to child and adolescent mental health in the school curriculum (e.g., self-harm, self-regulation, problem-solving skills).

*Many schools don’t even have counsellors, some [schools] fall on chaplains, yet the student numbers increase every year […] So, I think, periodically, GES must organise conferences, seminars and short in-service training for us [teachers] on the mental health matters of students, because at the [teacher] training college, there is nothing specifically on adolescent mental health, let alone adolescent self-harm or the suicides we’re now witnessing. GES must also put specific topics in the syllabus so that we can teach the students, for example, something like emotion management or life skills and other things. If that happens, the syllabus at the teacher training colleges must also [be] revised to accommodate these changes, so that teachers who come out [of training college] will have the knowledge and skills to help our students […] (M4, teacher, male).*

Although the GES representative concurred with the suggestion, his response did not reflect a strong sense of institutional commitment towards potential adoption of the suggested changes anytime soon. He intimated that the adoption of the suggestion by the GES would be challenging.

*The suggestions are in order, I think I subscribe to that, because, whether we like it or not, emotional and mental health issues affecting our children and students have become important these days, and we hear in the media of students committing suicide. I believe there are some general lessons already in the syllabus which touch on some of these issues, but they are not very detailed or specific. But given the exigencies and the realities around us now, I think we’d have to take a second look at the syllabus in our schools and also [in] our teacher training colleges and universities of education… But, you see, the challenge is that at GES, we implement policies; the Ministry of Education makes the policies for us and we implement. So, to make this kind of significant changes, it must be a collective thing – parents, teachers, the Ministry [of Education], GES, and the general society must accept that this is the way to go, else we cannot make any headway (M2, GES Representative, male).*

Similarly, the head of charity facility interviewed indicated that.

*[…] there are some policy documents and guidelines, for example, the Children’s Act, the Home Management Standards, and a few others which guide what we [charities] do, but specifically on the mental health of street children and young people, no. There is nothing concrete. As far as I know, there is only something on child health, which is mainly medical issue, but not mental health. You’d usually read a general thing like, ‘ensure that the children are psychologically and mentally well’. I mean, this is very vague, because there is nothing about what specific actions to take to ensure that these young people are mentally and psychologically well […] I know schools also don’t have anything on student mental health. So, we need something more concrete from the Department of Social Welfare (F2, female, head of charity facility).*

The Department of Social Welfare representative supported the suggestion and acknowledged the need for a child-and-adolescent mental health policy.

*The truth is that, there is nothing! At the moment, [we are] working on a policy document for the aged … However, for our kids, really, we never thought about that, the mental health aspect. I think it’s an oversight on our part. But generally, when you look at the whole system, mental health is less priortised. The other issue is that the Mental Health Act seems to pay more attention to hospital care, rather than community-based care. Can’t we find a way to train people to provide something like first aid mental healthcare within the community and then refer the serious issues to the hospitals? (M6, male, Department of Social Welfare Representative).*

## Discussion

This study represents the first research effort from Ghana at exploring the views of key adult informants regarding self-harm in adolescents. Some of the adults downplayed and rhetorically distanced themselves and their adolescents from self-harm, although they agreed that self-harm in adolescents is generally a hidden behaviour, and presently represents an issue of public concern in Ghana. Adult accounts of the motives and primary circumstances leading up to the onset and repetition of self-harm in adolescents were similar to those of adolescents with personal experience – more along the lines of social interactions with others, moral standards, and familial relationships, with little emphasis on individual level experiences (emotional states and thoughts) [8].

The respondents expressed a lack of competence and confidence in offering early support. Thus, the school staff identified their need for training, school mental health promotion protocols and curricula changes.

Although the participants in this study acknowledged the problem of adolescent self-harm, they engaged in rhetorical distancing in their responses to questions about self-harm by the adolescents they work with or live with. Interestingly, in our recent interview study with young people in Ghana, adolescents with self-harm histories (particularly in-school adolescents) acknowledged and identified their self-harm as a means of enacting taboo emotions and contestations in the family, and interpreted their self-harm as selfish, religious transgression, and as a social injury to significant others [8]. Most of the adolescents self-harmed in secret and did not seek help from parents or formal support sources [8]. Adolescents self-harm is labelled and wrongly judged as a reflection of poor or failed parenting [21–24]. Thus, it is plausible that some of the participants in the current study engaged in rhetorical distancing as a way of avoiding being judged by others – including researchers of self-harm – as poor parents, for failing to notice self-harm in their adolescents.

Given the highly stigmatised nature of self-harm and suicidal behaviours in Ghana [10], the posture of rhetorical distancing plausibly represents an effort by the participants to prevent themselves, together with their institutions, families, and adolescents from being stigmatised. Within the sub-Saharan African context this finding is not particularly surprising, as previous studies, for example, from Uganda, have shown that family and community members tend to adopt distancing as a symbolic cleansing ritual and a social practice of escaping social stigma related to suicidal behaviours [25]. Parents adopt rhetorical distancing to insulate their adolescent children from common youth risky behaviours (e.g., alcohol and substance use, sexual promiscuity) which are socially unacceptable and stigmatised [26]. Evidence from a recent systematic review and meta-ethnography of qualitative research on the role of schools in children and young people’s self-harm and suicide shows that school staff tend to place adolescents who self-harm into an “other” category, while distancing themselves from this “other” category; some school staff acknowledge the problem of self-harm in students, but view it as existing in other schools, among other students [27].

Rhetorical distancing can lead to invalid research findings, as participants seek to provide guarded and socially desirable responses to project their families, communities or schools as safe environments for young people. Also, this notion of “othering” and posture of distancing can decrease the opportunities for the detection of adolescent self-harm and prioritisation of intervention and prevention efforts [27]. However, in our study, the ‘insider’ status of the interviewer (EQ) and his ability to speak all relevant languages gave us the best opportunity one could hope for to overcome the challenge of distancing.

The emphasis on family relationships, social roles, morals, and context-specific factors in the narratives is consistent with the construction of the self and meaning-making system in collectivistic societies including Asia and Africa [28–32]. In these societies, collective norms, community characteristics and other-centred social roles and interactions, rather than individual specific characteristics and experiences constitute the frame of reference in the construction and presentation of the self and meaning-making in daily life [29–31].

Although many countries across Africa are witnessing social changes through Westernisation, media, and formal classroom education, which emphasise the freedom to exercise fundamental human rights, independence, assertiveness and individuality, most families and societies within the continent are still deeply rooted in patriarchy, with strict adherence to rules guiding traditional power relationships [33–35]. Young people who break the rules of obedience and respect and social comportment are often punished to deter repetition, save the honour of the family and protect traditional power relationship [34, 36].

Available evidence suggests that adults (including teachers, parents, and health professionals) who show positive, non-judgmental attitudes towards young people who (are at risk of) self-harm tend to positively influence future help-seeking intentions of these young people [27, 37–40].

Our respondents showed a serious effort at understanding the problem of self-harm in young people, with no indication of dismissive or critical attitudes. Even so, they - particularly the school staff - talked about their lack of competence and confidence in offering support to young people who (are at risk of) self-harm. This is consistent with recent evidence from South Africa and some high-income contexts where teachers reported lack of professional knowledge and skills in performing their role as frontline staff to offer mental health support to students who (are at risk of) self-harm [41–44]. Thus, as expected, the school staff and social workers in the present study suggested the inclusion of adolescent mental health issues in the social work and teacher training curricula and as part of social workers’ and teachers’ continuing professional development training programmes. Among other things, such in-service and pre-service staff training should address the fear related to the misconception that talking about self-harm or suicide ‘places the idea to try out the behaviour into the heads of young people’ [45], and the need to avoid separating self-harm neatly into ‘suicidal’ and ‘non-suicidal’ [46–49]. This suggestion of stakeholder training is imperative, as studies have shown that the training of key stakeholders and adult gatekeepers is critical to the supportive roles these adults play towards improving the mental health of children and adolescents [50–53].

Relatedly, the school and charity facility staff in the present study expressed a strong need for having well-articulated and clear protocols for responding to adolescent self-harm. Evidence from recent primary studies, systematic reviews, Delphi studies, and recommendations by key position papers have underscored the importance of schools and child and adolescent-centred institutions having protocols for responding to self-harm in young people [54–57]. “The advantage of having a written protocol is that staff know how to respond to self-injury systematically and strategically” [58].

The school staff in the present study suggested the inclusion of child and adolescent mental health issues (including adolescent self-harm) in the school curricula. Such adolescent mental health literacy promotion content could, among other things, focus on issues related to life skills development, emotion regulation, provision of peer support, and help-seeking. Emerging evidence from high-income contexts shows that school-based mental health promotion programmes lead to increased mental health awareness and favourably influence help-seeking behaviours among young people [59–62].

Some of the teachers in the present study also suggested that teachers’ roles should be redefined to include the provision of social care to students, to enhance teachers’ response to adolescent self-harm. There is an acute shortage of mental health professionals in schools in Ghana [63, 64]; where available, the school-based mental health professional is over-burdened with the simultaneous roles of teaching students and offering professional mental health services and support to both students and school staff. Hence, with the right pre-service and in-service staff professional training, some of the participants in this study believe that redefining the roles of teachers to include social care (e.g., identifying signs of self-harm, giving teen mental health first aid, providing additional onsite counselling, and signposting further support available to at-risk students) would help augment the efforts of school-based mental health professionals.

In a previous study from Israel on managing school violence, teachers who perceived and were willing to handle school violence as part of their core roles presented a significant positive influence on reducing school violence than teachers who perceived playing such roles as voluntary [65]. However, further evidence is needed for the finding in the present study; future studies could consider examining the endorsement and attitudes towards this suggestion (of adding social care to teachers’ roles) among a large sample of teachers in Ghana. The evidence from such future studies could help explore the boundary between the performance of core academic roles and the provision of social care by teachers; the evidence could also help address the potential concern of this suggested additional role creating an extra burden for teachers in schools that are acutely understaffed but with larger class sizes and increasing student populations [66].

### Strengths and limitations of study

As evident in our systematic review, the available qualitative studies exploring the first-person accounts of adolescent self-harm in sub-Saharan Africa have mainly been conducted in South Africa [5]. Thus, the present study represents the first from Western sub-Saharan Africa and Ghana, to document qualitative evidence on the views of key adult informants on self-harm in adolescents.

The findings from our study may be relevant to both the Ghanaian situation and the situation in other countries within sub-Saharan Africa. The contextual knowledge and practices related to socio-cultural norms and value systems, family life, education, and street living are more similar than different within and across countries in sub-Saharan Africa [30]. Universally, the evidence of this study is consistent with findings from recent global systematic reviews and meta-syntheses of evidence from primary (qualitative) studies in the area [52, 67]; the present study supports the observation that self-harm in young people is a global public health challenge [68].

Nonetheless, the reliability of the accounts of the participants in this study cannot be fully guaranteed. Even though attempts have been made throughout the analysis to explore the meanings of the results beyond the face value of the views of the participants, there is the need to accept the interpretations of the findings with caution. For example, we cannot know how much adults *are* aware of adolescent self-harm but choose not to acknowledge it. Also, the criminalised [14], socio-culturally proscribed status, tabooed and stigmatised nature of self-harm in Ghana [10, 11] might have created the tendency for some participants to misrepresent their lived events in guarded and socially desirable ways, engage in non-disclosure, and use other impression management strategies in the interview context [14].

## Conclusions

Although the adults we interviewed about young people who self-harm tend to downplay the scale of the problem, their attitudes towards those affected are not for the most part negative or dismissive. They see themselves as having a role in identifying those at risk and see the organisations in which they work as having a role to play in responding to individual young people in need. These are encouraging findings that point to at least one strand of a policy in Ghana for addressing the problem of self-harm in young people.

## Data Availability

The datasets used and/or analysed during the current study are available from the corresponding author on reasonable request.

## Abbreviation

GES: Ghana Education Service
WHO: World Health Organization

## Footnotes

1 The cross-sectional questionnaire survey and the present interview study adopt the definition of street-connected adolescents provided by Ghana’s Department Social Welfare and collaborators: a young person who is aged between 10 and 25 years, is born on the street and lives with parent(s) on the street; migrated to the street; or is an urban poor child or street mother who survives working in the street. Department of Social Welfare, Ricerca e Cooperazione, Catholic Action for Street Children, Street Girls Aid: **Census on street children in the Greater Accra region, Ghana**. Accra, Ghana: DSW; 2011.

2 The idea that adolescent self-harm is caused by the manipulations of unseen evil forces.

3 “Ashawo” is the local term in Ghana used to mean prostitute or having a promiscuous lifestyle.

4 Name of university in Ghana anonymised.

5 In Ghana, “pure water” is the local term for filtered water in sachet. Although it is sold in stores, it is predominantly sold to commuters, passengers, and pedestrians by sellers who walk along the streets in traffic jams.
